# Trends in the Use of Anxiolytics in Castile and Leon, Spain, between 2015–2020: Evaluating the Impact of COVID-19

**DOI:** 10.3390/ijerph18115944

**Published:** 2021-06-01

**Authors:** Miryam Sánchez Díaz, María Luisa Martín-Calvo, Ramona Mateos-Campos

**Affiliations:** 1 Faculty of Pharmacy, University of Salamanca, 37008 Salamanca, Spain; 2 Department of Physiology and Pharmacology, University of Salamanca, 37008 Salamanca, Spain; marisam@usal.es; 3 Department of Preventive Medicine and Public Health, University of Salamanca, 37008 Salamanca, Spain; rmateos@usal.es

**Keywords:** drug use, anxiolytics, COVID-19

## Abstract

Anxiolytics (N05B) are one of the most widely used pharmacological groups. This study aimed to analyze the progression of the consumption of anxiolytics (ATC classification: N05B) dispensed in pharmacies in Castile and Leon, Spain, from 2015 to 2020, with a special focus on the possible impact of COVID-19 on the use of these drugs. A quantitative-qualitative analysis of usage was carried out using the total number of packs and the packs per 1000 inhabitants. Overall, the use of anxiolytics grew by 14.41% during 2015–2020. The most commonly used drugs were the short-acting benzodiazepine derivatives lorazepam (whose use increased by 15.18%) and alprazolam (whose use increased by 21.40%), and the dispensing of the long-acting derivative diazepam increased the most, by 31.83%. Anxiolytics consumption increased significantly in 2020 and peaked in March. The pattern of use remained the same in 2020. The consumption of anxiolytics has continued to increase in Castile and Leon over the last six years. The COVID-19 pandemic situation affected the dispensing of these drugs, causing a sharp increase in prescriptions, especially during March, when the confinement of the population was initiated.

## 1. Introduction

Promoting mental health is a major public health concern regarding prevalence, disease, and disability burden. It is, therefore, one of the United Nations Sustainable Development Goals for 2030 [1].

Anxiolytics (N05B) are among the most widely used pharmacological groups in Spain where their use is even greater than the European average [2,3]. Although they are mainly applied to anxiety disorders, they are also administered to treat muscle contractures, neurovegetative dystonia and epileptic seizures [4].

The prevalence of chronic anxiety ranks tenth among people over 15 years of age, accounting for 6.7% of the population [5]. In 2017, packs of benzodiazepine derivatives (N05BA) were third among the chemical subgroups with the highest consumption in Spain (52,634,800 packs) [6].

These active drugs can generate tolerance and dependence, making it necessary to prolong treatment using increasingly higher doses, despite the risks of long-term use [7]. Thus, it is often difficult to decide when treatment should cease. Moreover, there is evidence that the use of benzodiazepines entails significant health risks for the population, and even their effectiveness has been questioned [8].

Since the 1990s, an annual increase in the consumption of anxiolytic drugs has been observed in Spain [9,10]; however, during the last three years (2017–2019), this trend has been on the decrease [2]. The present work aimed to analyze the progression of anxiolytic consumption dispensed by pharmacies in the Spanish region of Castile and Leon. It is the 3rd region of Spain with the oldest population and the second with the most deaths per 1000 inhabitants due to COVID-19. The study is carried out during the years ranging from 2015 to 2020. Special attention was paid to the possible impact of COVID-19 on the use of these drugs.

## 2. Materials and Methods

A descriptive study was conducted to dispense anxiolytics (therapeutic group N05B) by random pharmacies during 2015–2020. Data were obtained through the company IQVIA (Madrid, Spain), which is comprised of 620 pharmacies in Castile and Leon. According to generic and official brands, these pharmacies report data weekly regarding the total number of packs sold. These data were collected and extrapolated to include the total number of 1627 pharmacies within the autonomous community. We assumed that dispensing activity was equivalent to prescription and consumption since these types of drugs are only dispensed by medical prescription. The available data are the annual consumption by active substances in 2015 and the monthly consumption by active substances during 2016–2020.

The population data for each year studied (2015–2020) were obtained from the population census of Castile and Leon. This information is produced annually by the National Institute of Statistics [11].

Data are expressed as pack per 1000 inhabitants for years 2015–2020. To obtain a value for the consumption of packs per 1000 inhabitants, the total number of packs consumed was divided by the population size of Castile and Leon and multiplied by 1000. The percentage of the use of each active drug out of the total group was also obtained.

The anxiolytics considered in this analysis are alprazolam, bromazepam, clorazepate, diazepam, hydroxyzine, ketazolam, lorazepam and lorazepam pivalate. The data on the consumption of bentazepam, chlordiazepoxide, clobazam, clotiazepam, diazepam/pyridoxine/thiamine association, halazepam and pinazepam are included in the overall rate of consumption. Still, they have not been described in detail in the Results section as they presented values of less than 1% of use within the group.

Increases in consumption were calculated by subtracting packs per 1000 people in the final year from the value calculated for the first year. This difference was divided by the value of the first year and expressed as a percentage.

The SPSS Statistics program (Version 26. IBM. Armonk, NY, United States of America) has been used to carry out the statistical analysis. The Pearson correlation coefficient (r) was calculated as a linear correlation between two variables (X, Y). It ranges from −1 to 1. A value of 1 implies that a linear equation describes the relationship between the variables perfectly, with all data points lying on a line for which X increases as Y increases.

## 3. Results

**Figure 1:**
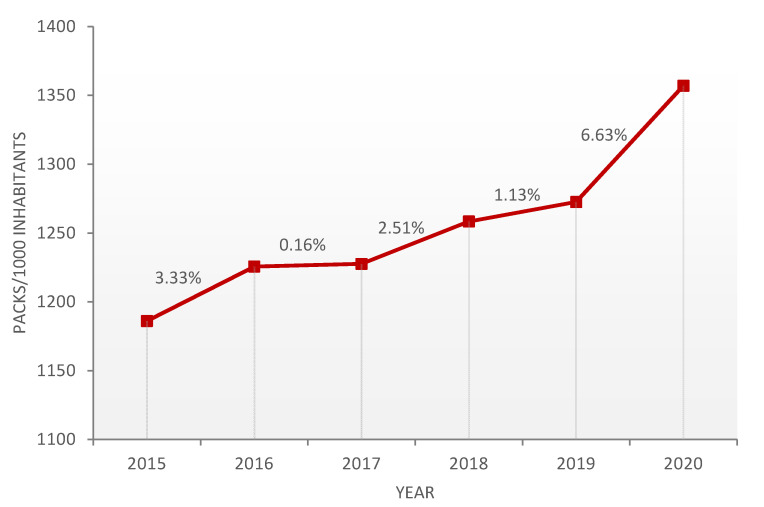
Progression of the consumption of anxiolytics by calculating annual variations.

The use of anxiolytics dispensed in pharmacies increased by 14.41% in Castile and Leon during the period ranging from 2015–2020, which equates to the increase of 1186 packs/1000 inhabitants to 1357 packs/1000 inhabitants. The Pearson correlation coefficient is 0.939. Consumption was found to increase annually throughout the period, although it remained stable in 2016 and 2017 (Figure 1).

Table 1 shows the progression in the increase in the consumption of the nine most commonly used anxiolytics out of a total of 15 studied. Lorazepam was the anxiolytic most consumed, although, in recent years, the sale of packs/1000 inhabitants did not increase as much as those of other anxiolytics, only 15.18%, which was 16.65% less than the increase in the consumption of diazepam, whose consumption increased by 31.83%. The use of bromazepam remained stable during 2015–2019 and increased in 2020.

In 2020, two anxiolytics, lorazepam and alprazolam, accounted for 65.04% of the total number of packs/1000 inhabitants prescribed. If bromazepam, diazepam and clorazepate are included, the cumulative percentage of use increased to 94.13%. Of the total 15 anxiolytics studied, these five drugs account for almost all the anxiolytics consumed thus complying with the Pareto Principle.

Although the amount of lorazepam consumed increased over the study period, its percentage of use, or market share, declined between 2015 and 2018 (34.18%–33.84%) but increased in 2019 (34.17%) and 2020 (34.41%). Alprazolam was the only one that maintained its increase in consumption and in the percentage of use throughout the study period (28.87%–30.63%). The percentage of use of diazepam grew up from 8.48% to 9.99% between 2015–2019 but decreased in 2020 (9.77%). The market share of bromazepam came down from 14.75% in 2015 to 13.78% in 2019 but slightly higher in 2020 (13.95%). The dispensing of clorazepate decreased by 3.06% and its rate of use also declined annually from 6.34% until 5.37%.

**Figure 2:**
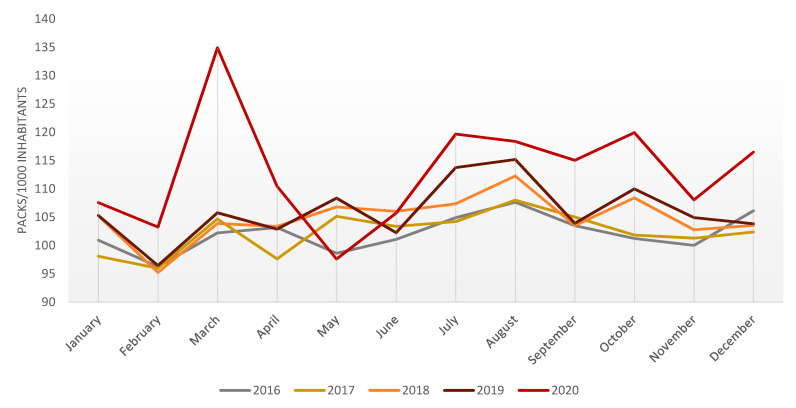
Monthly progression of the consumption of anxiolytics 2016–2020.

If we look at the monthly progression of the use of anxiolytics, there was a peak in consumption in March 2020, which increased by 30.65% compared to the previous month. The trend in consumption is similar to previous years, decreasing in February and increasing in March. Still, in 2020 the decrease in February is less pronounced, and the increase in March is much greater. In May, the trend of the last three years had been upwards but in 2020 there is a decrease in consumption below the four previous years. In June it increased again and remained above the use of previous years until December, following the same monthly trend of ups and downs as in the two previous years (Figure 2).

**Figure 3:**
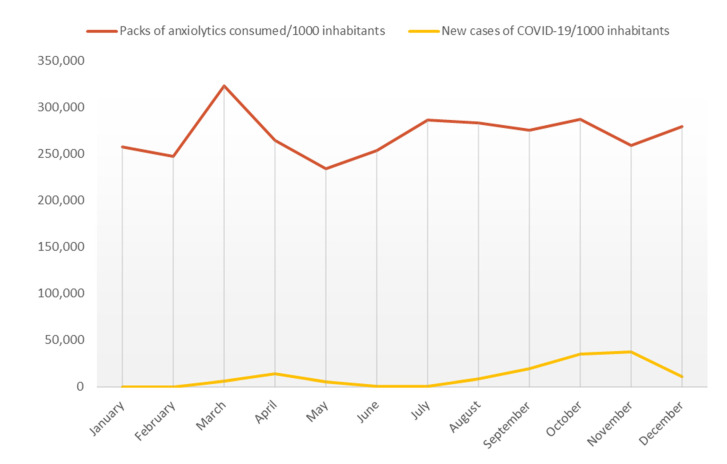
Progression of the new cases of COVID-19 per 1000 inhabitants in Castile and Leon in comparison with number of packs of anxiolytics consumed per 1000 inhabitants in Castile and Leon.

If we study the statistical relation between the number of new COVID-19 cases in Castile and Leon each month [12] with the evolution of the monthly consumption of anxiolytics using SPSS, this results in a Pearson correlation coefficient of 0.133. The correlation coefficient ranges from −1 (inverse relationship) to 1 (direct relationship). Hence, a value of 0 or close to it implies no linear correlation between the variables, as in this case (Figure 3).

The sale of all anxiolytics included in this study increased significantly during March compared to the previous month: Alprazolam increased by 33.20%, bromazepam by 44.05%, clorazepate by 30.97%, diazepam by 15.54%, hydroxyzine by 19.25%, ketazolam by 23.54%, lorazepam by 28.21% and lorazepam pivalate by 38.47%. All drugs follow the same pattern as the total amount of anxiolytics consumed in 2020 (Figure 2), which is more dramatic in the case of the most dispensed drugs, lorazepam, alprazolam, bromazepam, diazepam and clorazepate, which remained the same as in the 2015–2019 study period and with the same distribution.

**Figure 4:**
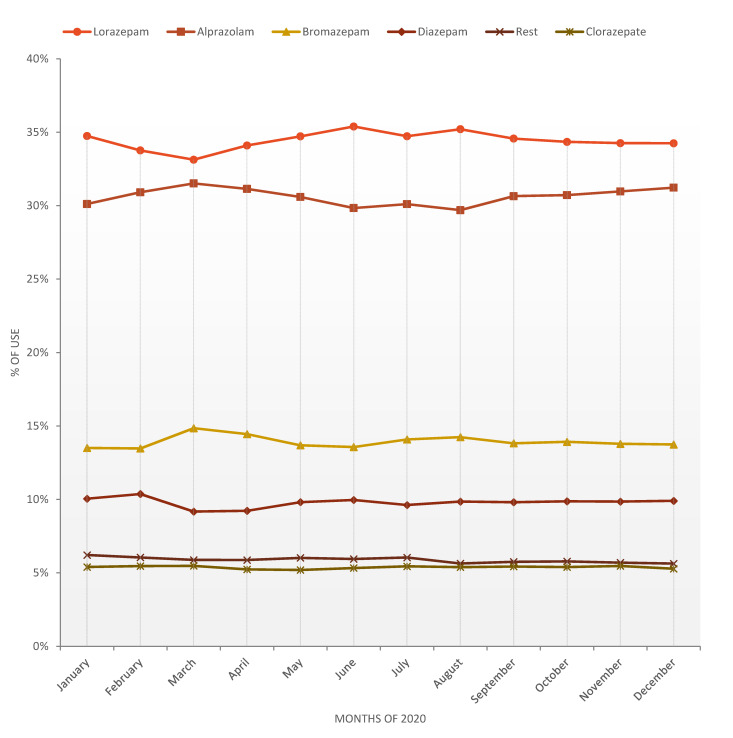
Progression of the use of anxiolytic drugs as a percentage in 2020.

The rate of use of lorazepam, which was the best seller during the entire period, reached its minimum in March and then recovered its market share until reaching its maximum in June. The progression of the percentage of use of Alprazolam was observed to be the inverse of that of lorazepam, although its consumption pattern in packs was the same. The percentage of the use of diazepam also declined in March despite a general increase in consumption over the rest of the months (Figure 4).

## 4. Discussion

The data from this study show that the consumption of anxiolytics in Castile and Leon has increased during the last six years, most significantly in 2020, although in Spain, on the whole, it decreased during 2015–2019. However, it also increased in the last year, from 55.25 DHD to 57.36 DHD, a value that had not been reached in the previous ten years [2].

The most commonly used anxiolytics were lorazepam and alprazolam. According to the Pareto diagram, their cumulative percentage of consumption in 2020 accounts for more than half of the total, which implies that these are the most frequently prescribed drugs. In the same period, these two drugs were also the most consumed in Spain. The use of alprazolam increased notably in Castile and Leon, although its usage decreased considerably in the country 2015–2019, it also increased in 2020. In contrast, the use of lorazepam in Spain remained relatively stable (2015–2019) [2] but increased in Castile and Leon, especially in its pivalate form. Lorazepam is prescribed for the short-term treatment of intense states of anxiety and tension that limit the patient’s activity and subject him or her to significant stress and the treatment of insomnia [13]. Alprazolam is indicated for the treatment of generalized anxiety, anxiety associated with depression and anxiety disorders [14]. These are two short-acting benzodiazepine derivatives, which are quickly eliminated from the body and thus involve less risk of sedation and accumulation, but at the same time must be taken two or three times a day for the anxiolytic effect to be maintained.

We find bromazepam, diazepam and clorazepate in a second group consumed by order of half compared to the above-mentioned drugs. If we add these three anxiolytics to the previous two, the cumulative percentage of consumption increases to 94.13%.

Bromazepam is also a short-acting derivative. Its use seems to have remained stable in the community, although use in Spain decreased. Diazepam and clorazepate are long-acting benzodiazepine derivatives. Diazepam was the drug that experienced the greatest increase in use during the period studied. Its use also increased in Spain during the same period, unlike clorazepate, which progressively decreased both in the country and in the autonomous community [2,15,16,17].

In the last five years, increases in consumption have been between 0.16% and 2.51%, which is a big difference compared with the increase in the number of patients prescribed anxiolytics in 2020, being 6.63% more than the previous year, an indication that the COVID-19 pandemic is not just leaving the after-effects produced by the disease itself. In many countries, the decision was taken to confine citizens to their homes to prevent the spread of the disease and prevent health systems from collapsing [18]. The confinement and uncertainty affected the mental health of the population [19,20], as was expected by many experts [21,22,23,24,25]. Most people have considered the initial psychological impact to be moderate or severe, with many of those affected reporting problems [26] such as depression or anxiousness. These psychological effects have been observed in populations worldwide, such as the United States [27], Hong Kong [28], China [26,29] and Japan [30].

In Spain, a nationwide state of alarm was declared on 14 March 2020 [31] due to the coronavirus and initiated the start of the confinement of the population. It was not until 4 May 2020, that the de-escalation plan, which consisted of four phases, came into force to create a “new normal” that would arrive at the end of June [32].

On 26 April 2020, the children were allowed to go for walks. On 2 May, adults could go out for the first time to do individual sports or hang out with a partner. Time slots had been established to divide the population. Those under 14 years old could leave from 12 noon to 7 p.m. and those over 14 from 6 a.m. to 10 a.m. and from 8 p.m. to 11 p.m. People over 70 and dependent people could leave from 10 a.m. to 12 p.m. and from 7 p.m. to 8 p.m. On June 8, Castile and Leon entered phase 2, it was one of the last communities to enter this phase in which time slots were abolished, and public places with limited capacity began to open. On June 15, five of the nine provinces of Castile and Leon entered phase 3, allowing mobility between them. It was not until June 22 that Castile and Leon entered the “new normal”, allowing movement between regions.

If we compare these dates with monthly consumption, we observe a maximum peak in the consumption of anxiolytics coinciding with the beginning of the confinement in March. By contrast, the lowest peak in consumption was observed when the entire population was allowed into the streets in May (Figure 2).

Restrictive confinement in Spain has been associated with significant psychological consequences [33]. According to a survey, Spaniards have expressed, even if only occasionally, feelings of worry (88.7%), anxiety (71.1%) and depression (39.8%). These alterations have mainly been brought on, above all, by the unsettling situation of seeing empty streets and stores (75.1%), concern regarding the inability to have face-to-face contact and interactions with relatives, friends and neighbors (71.8%), concern about the inability to recover one’s life as it was before the pandemic (64.9%), fear of getting sick (54.6%) and the fear of losing their employment or the death of a family member (53.4%) [34].

Anxiety symptoms have increased by 168.6% since the beginning of the state of alarm in Spain [35], and 45.7% of the people surveyed reported having experienced an increase in general psychological suffering. The individuals receiving psychological care did so more frequently for stress and anxiety symptoms (75.7%) [36]. In addition, the first study on a population of Spanish children concluded that 89% of children presented behavioral or emotional alterations as a result of the confinement [37].

There is evidence that the psychological consequences of COVID-19 will not end with the pandemic but that a small section of the population will subsequently develop anxiety, mood disorders and post-traumatic stress disorder [33].

The incessant increase in the consumption of anxiolytics and the excessive duration of treatment, in many cases chronic, was already a cause for concern. However, planning and intervention are even more urgent at present due to the increase in the number of new patients. It is necessary to study the reasons for prescription and evaluate other non-pharmacological treatment options to control possible dependence. This will also ensure that treatments are as short as possible, respecting the recommendations of not exceeding 8-12 weeks of continuous treatment, including the phase of gradual withdrawal (4 weeks in treating insomnia with lorazepam) [12,13,14,15,16].

## 5. Conclusions

The consumption of anxiolytics has significantly increased in the last six years (r = 0.939), with a large increase in 2020 than the trend observed in previous years.

It is shown that there is no linear relationship between the trend in consumption and the number of cases of COVID-19 in Castile and Leon. Still, it is observed that the restrictive confinement meant an increase in consumption that relaxed to values below even from the previous four years in May 2020 when the exit for sports and walking was allowed.

The consumption pattern has not changed in previous years as the most consumed are still lorazepam and alprazolam.

Moreover, the increase in the number of patients requiring prescribed anxiolytics causes us to reflect on their appropriate use, which should be in conjunction with adequate specialized psychological care and, therefore, it requires the implementation of mental health services.

## Figures and Tables

**Table 1:** Progression of the anxiolytics dispensed in Castile and Leon (packs/1000 inhabitants) during 2015–2020.

Active Drugs	2015	2016	2017	2018	2019	2020	Increment *
Alprazolam	342	361	366	377	383	416	21.40%
Bromazepam	175	178	175	174	175	189	8.23%
Clorazepate	75	76	74	73	70	73	−3.06%
Diazepam	101	107	112	122	127	133	31.83%
Hidroxyzine	28	24	24	25	25	25	−9.05%
Ketazolam	24	25	24	24	24	24	−2.51%
Lorazepam	405	419	418	426	435	467	15.18%
Lorazepam pivalate	17	19	19	22	21	18	4.93%
Total N05B group	1186	1226	1228	1258	1273	1357	14.41%

* The increment was calculated using the values taken from 2015 and 2020.

## Data Availability

Data sharing is not applicable to this article as no datasets were generated.
